# The Best Code

**Published:** 1892-11

**Authors:** 


THE BEST CODE.—The bulk of the profession is feeling more and more every day that the best code is one that can be expressed in the fewest clauses, and that the individual or community is best governed which is governed the least, and that the unwritten law which governs gentlemen is all that is necessary for any educated gentleman in any calling.—*Charlotte (N. C.) Medical Journal.*

